# Genomic analysis of carbapenemase-producing *Enterobacteriaceae* in Queensland reveals widespread transmission of *bla*_IMP-4_ on an IncHI2 plasmid

**DOI:** 10.1099/mgen.0.000321

**Published:** 2019-12-20

**Authors:** Leah W. Roberts, Elizabeth Catchpoole, Amy V. Jennison, Haakon Bergh, Anna Hume, Claire Heney, Narelle George, David L. Paterson, Mark A. Schembri, Scott A. Beatson, Patrick N. A. Harris

**Affiliations:** ^1^​ School of Chemistry and Molecular Biosciences, The University of Queensland, Brisbane, QLD, Australia; ^2^​ Australian Centre for Ecogenomics, The University of Queensland, Brisbane, QLD, Australia; ^3^​ Central Microbiology, Pathology Queensland, QLD, Australia; ^4^​ Public Health Microbiology Laboratory, Queensland Health Forensic and Scientific Services, Queensland Department of Health, Coopers Plains, QLD, Australia; ^5^​ University of Queensland, Faculty of Medicine, UQ Centre for Clinical Research, Royal Brisbane & Women’s Hospital, QLD, Australia; ^6^​ Australian Infectious Disease Research Centre, The University of Queensland, Brisbane, Australia

**Keywords:** CPE, IMP-4, IncHI2, Queensland, genomics, whole genome sequencing, nanopore

## Abstract

Carbapenemase-producing *Enterobacteriaceae* (CPE) are an increasingly common cause of healthcare-associated infections and may occasionally be identified in patients without extensive healthcare exposure. *bla*_IMP-4_ is the most frequently detected carbapenemase gene in *Enterobacteriaceae* within Australia, but little is known about the mechanisms behind its persistence. Here we used whole genome sequencing (WGS) to investigate the molecular epidemiology of *bla*_IMP-4_ in Queensland, Australia. In total, 107 CPE were collected between 2014 and 2017 and sent for WGS on an Illumina NextSeq500. Resistance genes and plasmid types were detected using a combination of read mapping and nucleotide comparison of *de novo* assemblies. Six isolates were additionally sequenced using Oxford Nanopore MinION to generate long-reads and fully characterize the context of the *bla*_IMP-4_ gene. Of 107 CPE, 93 carried the *bla*_IMP-4_ gene; 74/107 also carried an IncHI2 plasmid, suggesting carriage of the *bla*_IMP-4_ gene on an IncHI2 plasmid. Comparison of these isolates to a previously characterized IncHI2 plasmid pMS7884A (isolated from an *Enterobacter hormaechei* strain in Brisbane) suggested that all isolates carried a similar plasmid. Five of six representative isolates sequenced using Nanopore long-read technology carried IncHI2 plasmids harbouring the *bla*_IMP-4_ gene. While the vast majority of isolates represented ﻿* E. hormaechei *, several other species were also found to carry the IncHI2 plasmid, including *Klebsiella* species, *Escherichia coli* and *Citrobacter* species. Several clonal groups of *E. hormaechei* were also identified, suggesting that persistence of *bla*_IMP-4_ is driven by both presence on a common plasmid and clonal spread of certain *E. hormaechei* lineages.

## Data Summary

All sequencing data used in this study has been uploaded to the sequence read archive (SRA): Illumina (SRX5557687–SRX5557766, SRX5557773–SRX5557782). Nanopore (SRX5557767–SRX5557772). Illumina raw reads from previously sequenced CPE are also publicly available under BioProject PRJNA383436 (Illumina raw reads SRX2999336–SRX2999345, SRX5578807–SRX5578812, SRX5578814). The complete sequence type (ST)90 *Enterobacter hormaechei* reference genome is available from NCBI: MS7884 chromosome (CP022532), pMS7884A plasmid (CP022533) and pMS7884B plasmid (CP022534). The draft assemblies (Illumina and Nanopore) used for all comparative analyses are available on Github: https://github.com/BeatsonLab-MicrobialGenomics/CREATEQ_CPE.

**Impact Statement**

Gram-negative pathogens belonging to the family *Enterobacteriaceae *, such as *Escherichia coli *, *Klebsiella pneumoniae* or *Enterobacter* species may acquire genes that encode carbapenemases; these enzymes diminish the efficacy of carbapenem antibiotics. Carbapenemase-producing *Enterobacteriaceae* (CPE) are difficult to treat, but have been historically rare in Australia. More recently, CPE have caused outbreaks within hospitals and are now a major infection control concern. Previous work has demonstrated *bla*_IMP-4_ to be the most common carbapenemase in Queensland, but little is known about transmission within the population. We prospectively used whole genome sequencing of CPE collected from the main public referral laboratory in Queensland. The dominant carbapenemase was *bla*_IMP-4_, mainly found in species within the *Enterobacter cloacae* complex, in association with a widespread IncHI2 plasmid. The majority of *bla*_IMP-4_ were carried on a very similar IncHI2 plasmid which was found to be present across different clonal groups of *Enterobacter* and other species such as *Escherichia coli *, *Klebsiella* or *Citrobacter* species. Other carbapenemase genes were infrequent and almost exclusively associated with healthcare exposure overseas. The persistence of *bla*_IMP-4_ in Queensland is probably maintained by both clonal expansion of certain *Enterobacter* lineages as well as a common IncHI2 transmissible plasmid.

## Introduction

Carbapenemase-producing *Enterobacteriaceae* (CPE) are a growing burden worldwide, and infections with these organisms are often associated with significant morbidity and mortality [1, 2]. The family *Enterobacteriaceae* includes bacterial species such as *Escherichia coli *, *Klebsiella pneumoniae* and the *Enterobacter cloacae* complex, which are responsible for the majority of infections caused by gram-negative bacteria [3]. CPE typically carry a variety of antibiotic resistance genes, leaving few effective treatment options [4, 5]. CPE transmission occurs most frequently in clinical environments [6]; however, community-acquired CPE are increasingly recognized, posing a great threat to public health [6, 7].

CPE genes endemic to certain geographical locations have been described, such as *bla*_KPC_ in the United States and parts of Europe [8, 9], *bla*_NDM_ in India and China [10], OXA-48-like carbapenemases in Mediterranean countries and the Middle East [9], and *bla*_IMP_ in Australia and the Asia–Pacific region [11, 12]. CPE may be maintained in the population by the transmission of carbapenemase genes circulating on plasmids or via clonal expansion [6]. Few studies have used whole genome sequencing (WGS) on large geographically related datasets, enabling in-depth analyses into the genomic context of carbapenemase genes and their associated plasmids [13, 14].

This was a prospective cohort study of patients admitted to any Queensland Health facility served by the state-wide microbiology laboratory network (Pathology Queensland), who were colonized or infected with CPE. The aim was to describe the clinical features of patients with CPE, define the range of CPE species, identify the dominant carbapenemase genes, and elucidate patterns of clonal or plasmid transmission using WGS.

## Methods

### Setting

Pathology Queensland is a network of 35 laboratories serving all public hospitals in Queensland, Australia. According to laboratory protocols, suspected CPE or carbapenem-resistant *Enterobacteriaceae* are referred to the central laboratory in Brisbane for further analysis.

### Isolate selection and CPE detection

Susceptibility testing was performed using Vitek 2 (bioMérieux), with minimum inhibitory concentrations (MICs) for meropenem also determined using MIC gradient tests (Etest; bioMérieux). Carbapenemase production was detected using colorimetric methods (RAPIDEC CARBA-NP; bioMérieux; or β CARBA; Bio-Rad) and chromogenic agar (chromID CARBA SMART Agar; bioMérieux). Any *Enterobacteriaceae* with a meropenem MIC >0.25 mg l^−1^ by Vitek2 or ≥0.125 mg l^−1^ by Etest [15], a positive colorimetric test for carbapenemase or growth on chromID CARBA SMART agar was submitted for molecular confirmation of CPE status using a multiplex PCR assay targeting NDM, IMP-4-like, VIM, KPC and OXA-48-like carbapenemase genes [16–19]. Any *Enterobacteriaceae* isolated between January 2014 and May 2017 with PCR confirmation of carbapenemase genes, or suspected carbapenemase production by phenotypic methods, was characterized further by WGS. Only a single CPE isolate per patient was included in the WGS analysis. However, additional CPE sequences were included if a different CPE species was subsequently isolated or PCR demonstrated the presence of a different carbapenemase gene as compared with the initial organism.

### Clinical data

Clinical variables of patients with CPE were collected from the electronic medical record. This included ward of admission at the time of CPE detection, admitting clinical service, body site of initial specimen with CPE, the Charlson Comorbidity score [20] at admission, and other significant risk factors for infection (end-stage renal failure requiring dialysis, solid organ or haemopoietic stem cell transplant, cytotoxic chemotherapy, monoclonal antibody therapy or other immunosuppression). The following were recorded if present within 12 months prior to CPE detection: any history of travel or healthcare exposure overseas, prior admission to hospital or regular healthcare exposure (e.g. haemodialysis), intensive care unit (ICU) admission, previous colonization or infection with an extended-spectrum β-lactamase (ESBL)-producing *Enterobacteriaceae *, methicillin-resistant *Staphylococcus aureus* (MRSA) or vancomycin resistant enterococci (VRE). The presence of any of the following was recorded if present within 1 month prior to CPE detection: surgical procedures, endoscopy, any episode of neutropenia and antibiotic exposure. Any directed antibiotic therapy for the CPE and its duration was recorded, as was the outcome (died or survived) up to hospital discharge.

### DNA extraction and sequencing

DNA was extracted from pure bacterial colonies after overnight incubation on horse blood agar at 37 °C, using the DSP DNA Mini Kit on the QIAsymphony SP instrument (Qiagen). Libraries were prepared using the Nextera XT DNA preparation kit (Illumina) and sequencing was performed on the NextSeq 500 (Illumina) with 2×150 bp chemistry, NextSeq Midoutput kit v2.5. Isolates for sequencing with the Oxford Nanopore MinION were plated on Luria Bertani (LB) agar and incubated overnight at 37 °C. DNA was extracted using a Qiagen DNeasy UltraClean Microbial kit (as per the manufacturer’s instructions). Library preparation for eight isolates was done using the 1D sequencing by ligation kit (SQK-LSK108) with the native barcoding expansion kit (EXP-NBD103) to multiplex all isolates on a single FLOW-MIN106 R9.4 flow cell. Sequencing on the MinION with local base-calling ran for ~40 h, generating 65 993 reads.

### Binning and quality control (QC)

Raw Illumina reads were checked for quality using FastQC and trimmed using Trimmomatic v0.36 [21], filtering for Illumina adapters, bases below Q10 and reads less than 50 bp in length. Raw reads were checked for contamination using Kraken (v0.10.5-beta) [22]. Nanopore reads were base-called locally using Albacore (v2.3.1) and demultiplexed using Porechop v0.2.3_seqan2.1.1 (https://github.com/rrwick/Porechop) with default settings. The resulting read bins were filtered using Japsa v1.5-11a (https://github.com/mdcao/japsa) for reads below Q10 and less than 2000 bp in length.

### Assembly

Trimmed Illumina reads were *de novo* assembled using SPAdes v3.11.1 [23] under default settings. *De novo* assembly metrics are provided in the Supplementary Data (available in the online version of this article). Isolates CQS2 and CQS52 were removed from the study as more than 10 % of the assembled genome was found to be <10× coverage and in contigs <100 bp. Filtered Nanopore reads were *de novo* assembled using Canu v1.7 [24] with default settings. CQS39 and CQS33 were removed from the long-read analysis due to low throughput and batch problems with the barcoding kit.

### Plasmid comparisons

Draft assemblies were compared to plasmid reference sequences using the nucleotide comparison tool BRIG [25]. Draft Nanopore assemblies were compared to the reference IncHI2 plasmid pMS7884A (GenBank: CP022533.1) using EasyFig [26]. Average nucleotide identity (ANI) was calculated using FastANI v1.1 [27]. The number of shared bases between the isolates and the reference IncHI2 plasmid pMS7884A was estimated by mapping trimmed-reads to the plasmid using Bowtie2 v2.3.4.2 (as implemented through Nesoni v0.132: https://github.com/Victorian-Bioinformatics-Consortium/nesoni) and calling all conserved regions using the Nesoni nway function with the ‘–all’ flag. All coding sequences (CDS) from the multidrug resistance (MDR) region in the reference plasmid pMS7884A were combined to create a non-redundant list of 48 CDS. These CDS were compared against isolate draft assemblies using Abricate (v0.8; https://github.com/tseemann/abricate; ‘–minid 90’, ‘–mincov 90’) to determine presence and absence of genes in this region (reference available on GitHub: https://github.com/BeatsonLab-MicrobialGenomics/CREATEQ_CPE).

### Sequence types (STs), antibiotic resistance gene profile and plasmid profiles

ST was determined using the tool MLST (v2.1; https://github.com/tseemann/mlst) against the filtered draft assemblies. Antibiotic resistance genes and plasmid incompatibility groups were also determined using the filtered draft assemblies and the tool Abricate (v0.8; https://github.com/tseemann/abricate) against the ResFinder [28], ARG-ANNOT [29] and PlasmidFinder [30] databases (last updated April 2018) using a minimum coverage of 90 % with 90 % nucleotide identity. Trimmed reads were also compared against the ARG-ANNOT database using SRST2 [31] to confirm results obtained through Abricate.

### Phylogenetic analyses

Parsnp (v1.2) [32] was used to create a phylogeny using the draft assemblies of all isolates against the *Enterobacter hormaechei* strain MS7884 chromosome (gbk: CP022532.1) under default settings (67 457 SNP positions). MS7884 was originally isolated in south-east Queensland and was chosen as the reference to represent the majority of isolates in this dataset, which were part of the *E. cloacae* complex. Metadata was added to the resulting tree (mid-point rooted) using Phandango [33]. SNP distances were determined by mapping trimmed reads from *E. hormaechei* isolates to representative draft assemblies for each ST using Bowtie2 v2.3.4.2 (as implemented through Nesoni v0.132: https://github.com/Victorian-Bioinformatics-Consortium/nesoni) (see Supplementary Data for reference genomes and ST groups). SNV (Single nucleotide variant) relationship matrices (Nways) produced by Nesoni were interrogated manually to remove low-confidence SNPs, specifically (1) those that were inconsistent between mapping and assembly of the same strain, and (2) those that had an ambiguous allele call (and therefore possible mismapping to a repetitive region in the genome). The relationship matrices were built both manually and using the Neighbor-Joining function (Hamming distance, Saitou–Nei criterion) in Phyloviz v2.0 [34] based on SNPs detected using Nesoni.

## Results

**Fig. 1.**
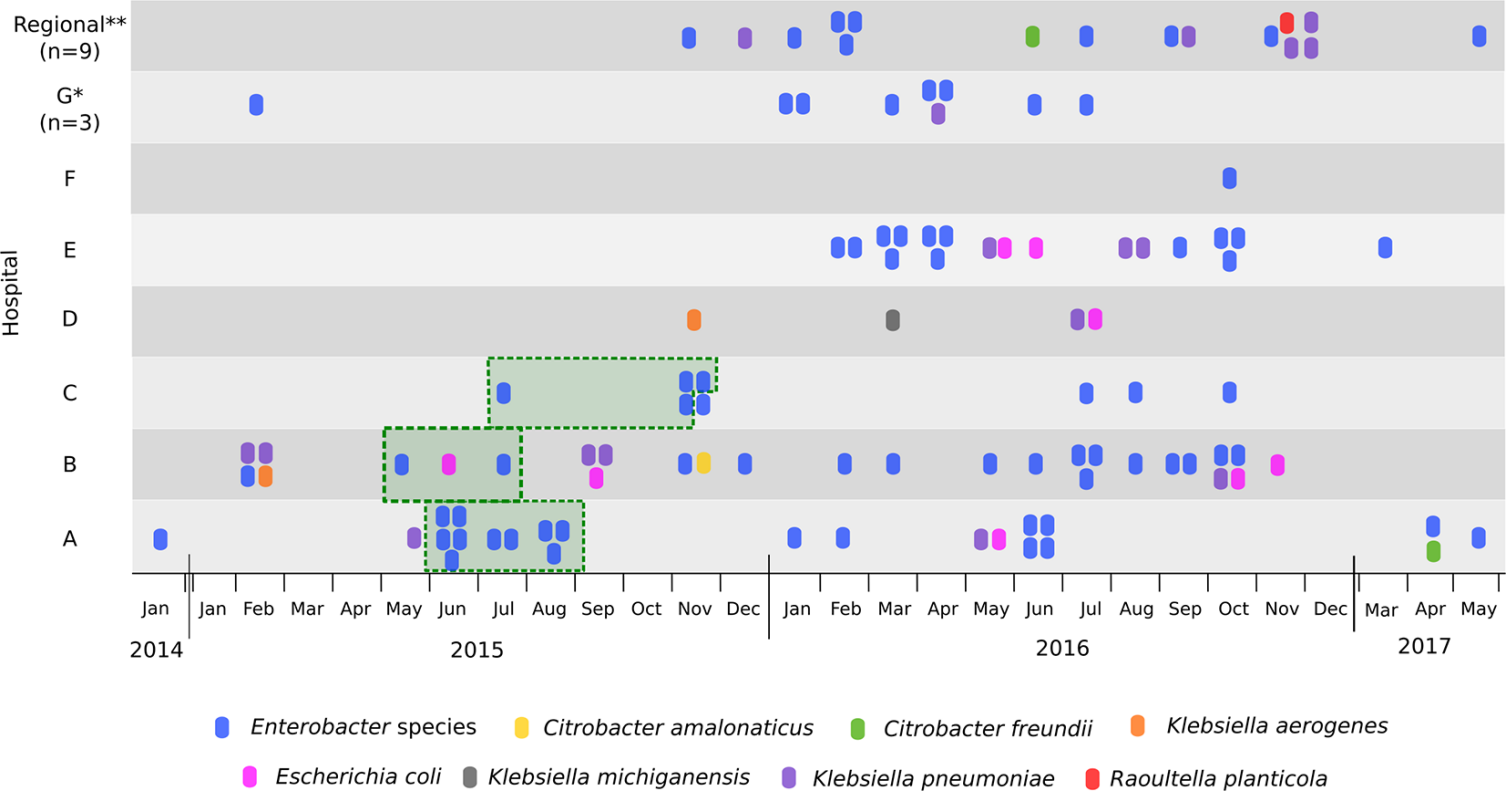
Timeline of samples from hospitals in Brisbane (Hospitals A–F) and Regional Queensland (QLD). Isolates in green boxes represent those related to the 2015 carbapenemase-producing *E. hormaechei* outbreak [34]. *Three hospitals located on the outskirts of Brisbane. **Nine hospitals from around Regional QLD. See Supplementary Data for a comprehensive list of isolates.

From 2014 to 2017, a total of 94 CPE from Queensland were identified from 81 patients (nine patients had more than one CPE isolate sequenced) (Fig. 1). During this period, 179 suspected CPE isolates were submitted to the central laboratory for PCR testing. Cumulative antibiogram data for all public hospitals served by Pathology Queensland in 2017 demonstrated that <1 % of all *Enterobacteriaceae* were resistant to meropenem. An additional four CPE strains cultured from endoscope surveillance samples were also included in the analysis, as well as nine CPE (from two additional patients) sequenced during an outbreak in 2015 [35], bringing the total number of strains to 107 from a total of 83 patients (Table S1, Supplementary Data). The clinical features of patients colonized or infected with CPE are summarized in Table 1. The mean age of patients was 63 years (range <1 to 96) and 54 % were female. Few patients (6/83; 7.2 %) had travelled overseas within the previous 12 months, all of whom had healthcare exposure [India (*n*=4), Greece (*n*=1), Kenya (*n*=1)]; 77 % were born in Australia. While the majority of CPE (85 %) were identified from the larger hospitals within the greater metropolitan area of Brisbane, a total of 18 hospitals across the state reported CPE cases. The most common primary sites of isolation included cultures from rectal swabs (42.2 %), urine (34.9 %), blood (10.8 %), sputum (9.6 %), intra-abdominal (3.6 %), wound (6.0 %), central nervous system (2.4 %) and osteoarticular (2.4 %) samples (note some patients had more than one positive sample site). A large proportion (36.1 %) of these were thought to reflect colonization only at the time of isolation. Hospital admission in the preceding 12 months was common, with a median number of admissions of two in the previous year (range 0–20); 28.9 % of patients had been admitted to an ICU within 12 months and 72.3 % reported a hospital admission within 1 month of CPE identification. The median Charlson co-morbidity score was 2 [interquartile range (IQR) 1–4, range 0–12]. Only 42.0 % of patients identified received any antibiotics for CPE infection (most commonly meropenem, fluoroquinolones, amikacin or colistin, often in combination), and 90.2 % survived to hospital discharge.

**Table 1.** Clinical characteristics of patients with CPE

Clinical variable	Level	Value
Patient age (years), mean±SD		63.4±17.0
Sex	Male	38 (45.8 %)
	Female	45 (54.2 %)
Region of birth	Australia/NZ	68 (81.9 %)
	Europe	12 (14.5 %)
	South Asia	1 (1.2 %)
	Pacific Islands	1 (1.2 %)
	Africa	1 (1.2 %)
Admitting service	Medical	33 (39.8 %)
	Surgical	33 (39.8 %)
	ED/ICU	13 (15.7 %)
	Other	4 (4.8 %)
Residence	Private home	50 (60.2 %)
	Nursing home	6 (7.2 %)
	Other residential care facility	1 (1.2 %)
	Inter-hospital transfer	26 (31.3 %)
No. of admissions in previous 12 months, median (IQR)		2.0 (1.0, 5.0)
ICU admission within 12 months		24 (28.9 %)
Any travel outside Australia with 12 months		6 (7.2 %)
Surgical procedure within past 1 month		37 (44.5 %)
Charlson score, median (IQR)		2.0 (1.0, 4.0)
MRSA within 12 months		3 (3.6 %)
VRE within 12 months		17 (20.5 %)
ESBL within 12 months		3 (3.6 %)
Immunosuppression		9 (10.8 %)
Any antibiotics within 1 month		62 (74.7 %)
CPE treated with antibiotics		34 (42.0 %)
Total duration of CPE therapy (days), median (IQR)		7.0 (3.0,13.0)
Outcome	Died in hospital	9 (10.8 %)
	Survived to discharge	74 (89.2 %)

*In three cases, CPE was likely to have contributed to mortality.

### Spread of *bla*_IMP-4_ mainly facilitated by IncHI2 plasmid in Queensland

**Fig. 2.**
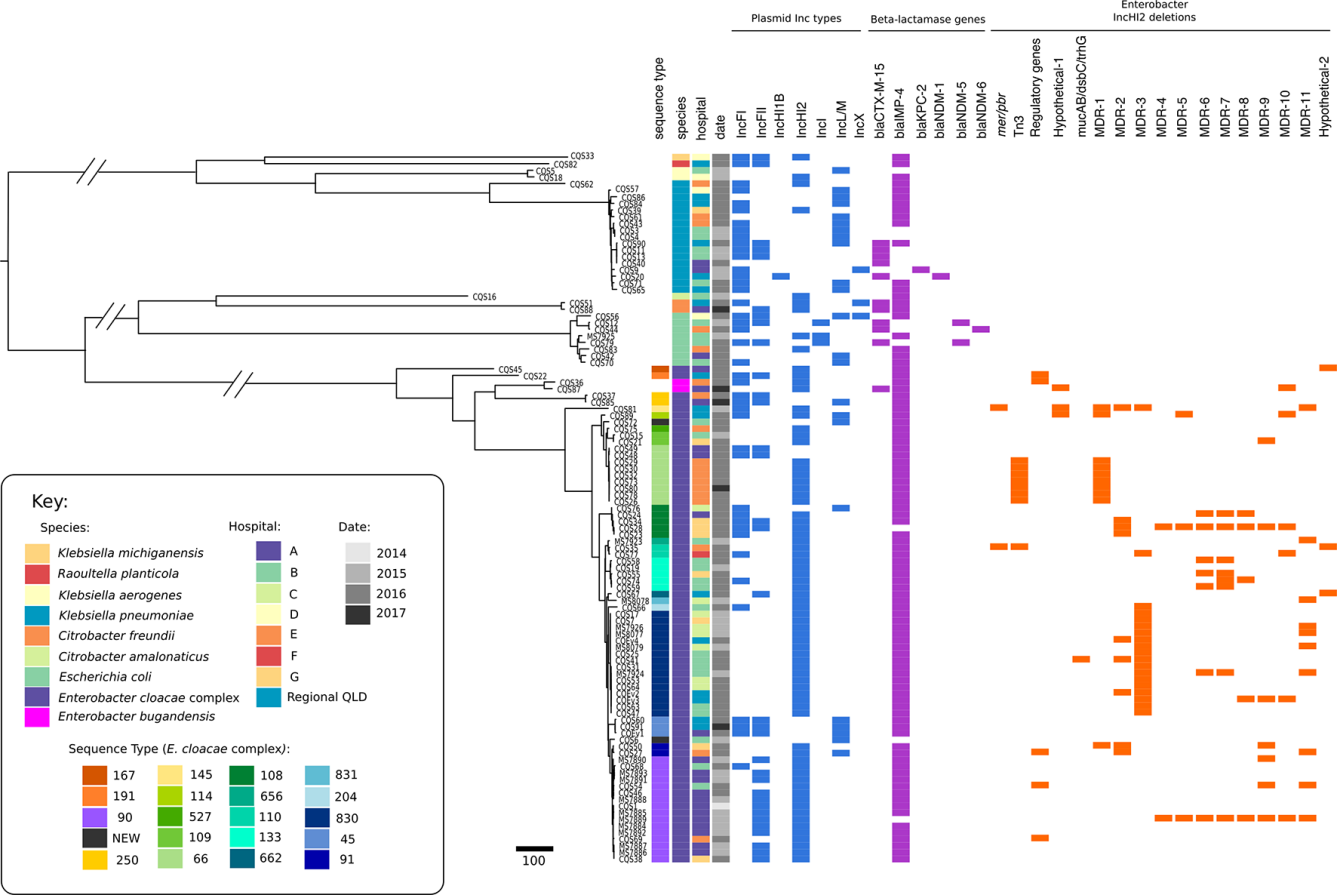
Phylogenetic tree of all isolates: the tree was built using 107 CPE collected between 2014 and 2017 using Parsnp v1.2 (default settings) against the reference genome MS7884 (GenBank: CP022532.1). Plasmid types and resistance genes shown here are a selection of the most prevalent types. The majority of isolates were found to be part of the *E. cloacae* complex, with a high prevalence of *bla*_IMP-4_ and IncHI2 plasmid types. Orange blocks represent missing plasmid regions identified by comparison of IncHI2 + *Enterobacter* isolates to the IncHI2 plasmid pMS7884A using BRIG (see Fig. S7 and Supplementary Data).

Most isolates recovered in this study were part of the *E. cloacae* complex (73/107; 68 %), representing more than 10 different STs (Fig. 2, Table S1, Fig. S1), the most common being ST90 (*n*=16) and ST830 (*n*=16). The collection also included *Enterobacter bugandensis* (*n*=2), *Klebsiella aerogenes* (*n*=2), *Citrobacter amalonaticus* (*n*=1), *Citrobacter freundii* (*n*=2), *Escherichia coli* (*n*=8), *Klebsiella michiganensis* (*n*=1), *K. pneumoniae* (*n*=17) and *Raoultella planticola* (*n*=1). The most prevalent carbapenemase gene was *bla*_IMP-4_, which was present in 87 % (*n*=93/107) of CPE (Figs S2–3). Other carbapenemase genes were only infrequently encountered, such as *bla*_OXA-48-like_ [*bla*_OXA-181_ (*n*=3), *bla*_OXA-232_ (*n*=1), *bla*_OXA-48_ (*n*=4)], *bla*_NDM-[1, 5, 6]_ and *bla*_KPC-2_. Several of these uncommon carbapenemase genes were related to recent travel (see Supplementary Results Section 1). Only one case of dual-carbapenemase gene carriage was identified [CQS20 (* K. pneumoniae *): *bla*_NDM-1_ and *bla*_OXA-232_].

Overall, the majority of isolates were found to carry an IncHI2 plasmid replicon (*n*=74/107, 69%). In a previous study we used Pacific Biosciences long-read sequencing to completely characterize an ~330 kb IncHI2 plasmid carrying *bla*_IMP-4_ that was linked to a nosocomial outbreak in Brisbane [35]. Nucleotide comparison of our CPE draft genomes collection to this reference plasmid (pMS7884A, GenBank: CP022533.1) found that the majority of IncHI2-positive isolates appeared to carry closely related plasmids (on average 83.2 % of the reference plasmid pMS7884A was conserved based on read mapping). This included 65 of 75 *Enterobacter* isolate genomes which exhibited greater than 98 % nucleotide identity across on average 87.2 % of pMS7884A (58–96.2 % range based on read mapping) (Fig. S7 and Supplementary Data). Conversely, species other than *Enterobacter *, such as *Escherichia coli* and *K. pneumoniae *, had a lower prevalence of this plasmid (9/32) (Fig. S8, Table S2). However, despite most lacking a pMS7884A-like plasmid, these non*-Enterobacter* isolates shared notable similarities in the gene content with the ~55 kb MDR region previously defined on pMS7884A [35]. Overall, non-* Enterobacter* species carried on average 35 % of the MDR region (based on comparison of non-redundant CDS regions). Interestingly, those that carried the *bla*_IMP-4_ gene carried twice as many CDS from this region compared to those that did not (24 % vs 48 % on average) (see Supplementary Data). These results suggest that *bla*_IMP-4_ has been mobilized via an integron or other transposable element from a pMS7884A-like plasmid.

Based on these findings, we propose that the prevalence of *bla*_IMP-4_ is mainly facilitated by the dissemination of a pMS7884A-like plasmid among a diverse range of *Enterobacter* STs around Queensland. The increased prevalence of certain STs among the dataset also suggests probable expansion of specific *Enterobacter* clonal lineages, such as ST90 and ST830 (Fig. 2).

### Long-read Oxford Nanopore MinION sequencing further resolves the context of *bla*_IMP-4_ in a range of bacterial species

**Fig. 3.**
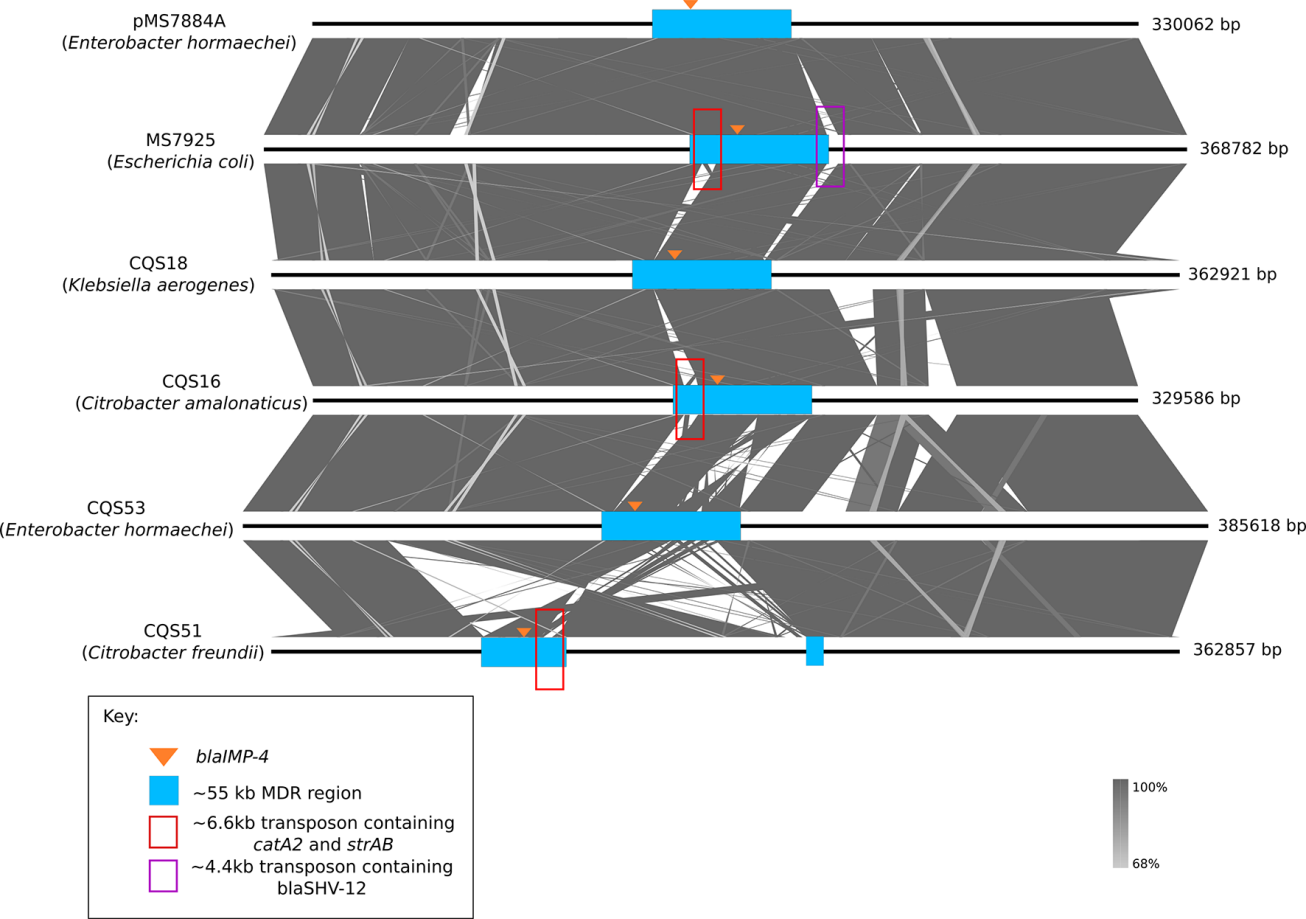
Arrangement of IncHI2 plasmids in a variety of *bla*_IMP-4_-positive species: several isolates of various species were subjected to Nanopore MinION sequencing to completely resolve their IncHI2 plasmids. *De novo* assembly and nucleotide comparison to the reference plasmid pMS7884A revealed very similar IncHI2 plasmids in all isolates. The ~55 kb MDR region (blue box) appeared conserved in all isolates, despite being interrupted by a large inversion in CQS51. Several losses or gains of small regions within the MDR region were observed, noted by the red and purple boxes.

To further understand the stability and context of the *bla*_IMP-4_ carbapenemase gene in other species, we selected six isolates from this dataset for long-read sequencing with Oxford Nanopore MinION. The six isolates represented *E. hormaechei *, *Escherichia coli *, *K. aerogenes *, *C. freundii* and *C. amalonaticus *. Plasmids carrying *bla*_IMP-4_ were assembled from the MinION long-read data for six isolates. Five of the six isolates all carried the *bla*_IMP-4_ carbapenemase gene on an IncHI2 plasmid with >98 % ANI to pMS7884A (isolated from an *E. hormaechei* strain in 2015) (Fig. 3). The ~55 kb MDR region was mostly conserved, except for a small ~6.6 kb region containing the *catA2* and *strAB* genes that was not present in CQS18 (* K. aerogenes *) and CQS53 (* E. hormaechei *). Isolate MS7925 (* Escherichia coli *) was found to have acquired an additional ~4.4 kb transposon carrying *bla*_SHV-12_ at the 3′ end of the ~55 kb MDR region. CQS53 contained a small inversion within the ~55 kb MDR region. CQS51 contained a large inversion that encompassed part of the MDR region and also the IncHI2 plasmid backbone (Fig. 3).

The additional *E. hormaechei* isolate, CQS89, carried both an IncL/M and an IncHI2 plasmid. The IncHI2 plasmid backbone was highly similar to pMS7884A, but the *bla*_IMP-4_ gene, as well as the In809 integron, were located on the IncL/M plasmid, flanked by IS*26* (Fig. S10). The IncL/M plasmid also carried an ~16 kb region with genes relating to tunicamycin, macrolide and chromate resistance.

### IncL/M plasmids as possible vector for *bla*_IMP-4_ and *bla*_OXA-48_ carbapenemases

The nine *Enterobacter* isolates that did not carry an IncHI2 plasmid, but retained *bla*_IMP-4_, were found to have IncFIB (*n*=8), IncFII (*n*=7), IncL/M (*n*=6) and IncR (*n*=1) plasmid types. As carriage of *bla*_IMP-4_ has previously been described on IncL/M-type plasmids in Australia [36], we compared all IncL/M-positive isolates to the previously described IncL/M plasmid pEl1573 isolated in Sydney, Australia (GenBank: NC_019368.1 [36]). Of 23 isolates, we found 10 that appeared to carry a very similar plasmid to pEI1573, including the *bla*_IMP-4_ gene within a large region comprising the majority of other resistance genes (Fig. S9, Table S3). Four isolates appeared to have retained the IncL/M plasmid backbone, but lost the entire resistance region, which was consistent with the lack of *bla*_IMP-4_ and other resistance genes. These four isolates [CQS3 (* K. pneumoniae *), CQS4 (* K. pneumoniae *), CQS5 (* K. aerogenes *) and CQS6 (* E. hormaechei *)] all carried a *bla*_OXA-48_ gene instead, suggesting an interchangeable resistance region on the IncL/M plasmid. All four isolates were collected from the same patient, demonstrating the transmissibility of this suspected *bla*OXA-48-carrying IncL/M plasmid to all species within this patient. The remaining isolates (*n*=9) appeared identical to pEI1573 except for the loss of an ~5 kb region containing ISCR1, *qnrB2* (responsible for resistance to fluoroquinolones), *qacEdelta1* (responsible for resistance to quaternary ammonium compounds) and *sulI* (responsible for resistance to sulphonamides).

### Analysis of *E. hormaechei* ST groups reveals possible long-term transmission

**Fig. 4.**
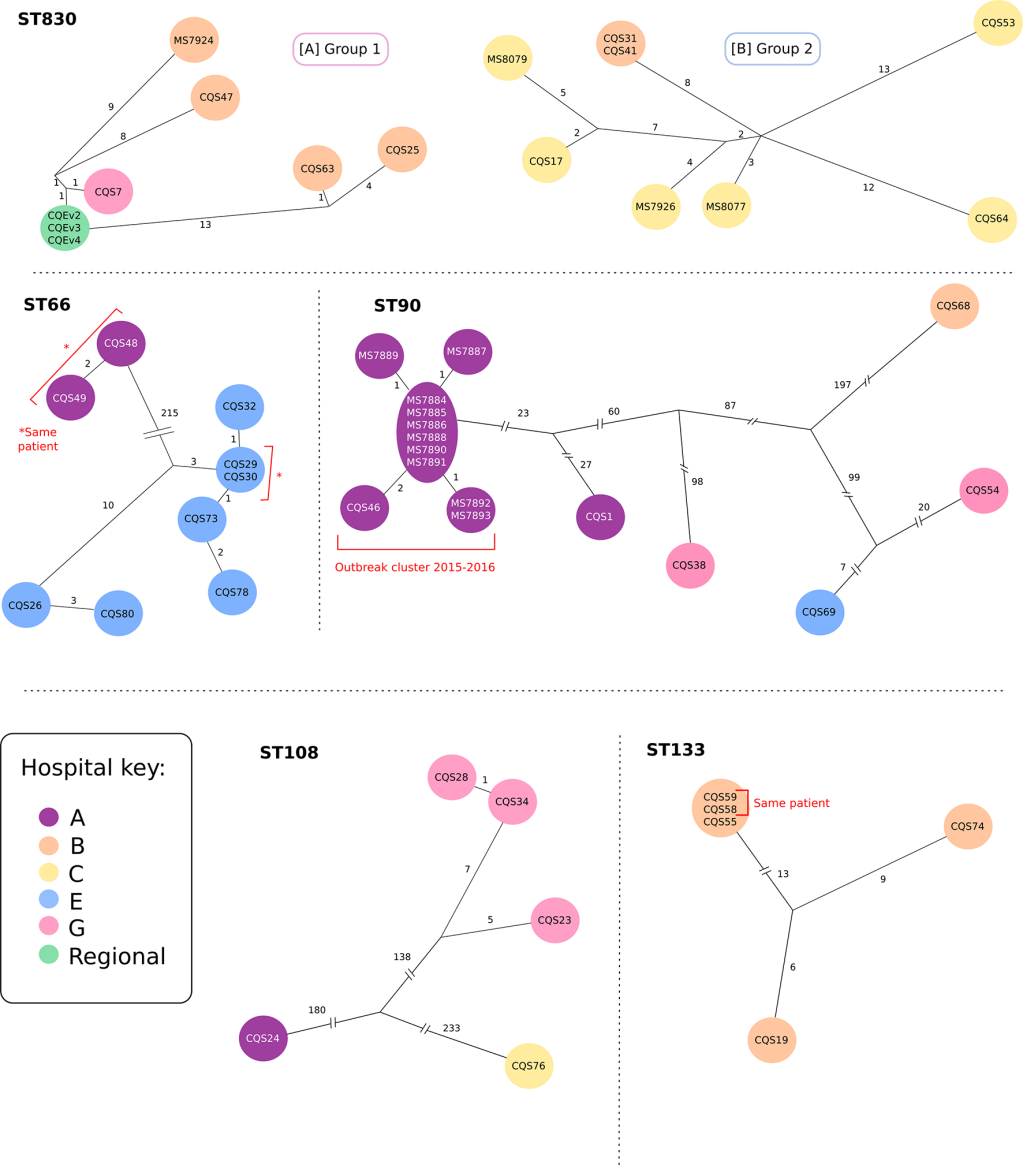
*
E. hormaechei* SNP relationships: *E. hormaechei* STs that had two or more isolates were analysed to determine their relatedness and any possible transmission events. Four ST groups (ST830, ST66, ST108 and ST133) were found to have evidence of recent and/or long-term transmission. Each figure represents core SNP differences between isolates within that ST group: isolates within the same circle are identical at the core genome level, while the black lines represent the number of SNPs different between isolates. Colours represent the hospital where the isolate was sourced. There was no evidence of transmission within the ST90 group outside of what has already been reported [35].

A number of ST830 *E. hormaechei* isolates were recovered from similar geographical locations, indicating a possible clonal relationship and ongoing transmission within the area. To investigate this, we analysed the SNP differences between *E. hormaechei* ST830 isolates, which revealed two distinct groups (Fig. S11). By mapping reads from the ST830 isolates to references within each group, we found that group 1 isolates were on average 6 SNPs away from the next closest isolate (range=2–11 SNPs), while in group 2 there were on average 11 SNPs distant (range=7–16 SNPs) (Fig. 4). Based on these SNP distances, it is unlikely that these strains are related by direct recent transmission. However, the clustering of similar strains primarily within two hospitals over a period of 18 and 13 months (for groups 1 and 2, respectively), as well as the identification of environmental isolates, suggests undetected transmission over a long period of time (Fig. 4).

To identify other possible transmission events, we analysed an additional nine *E. hormaechei* ST groups containing two or more isolates from different patients using the same read-mapping method as with the ST830 isolates (see Supplementary Data). Comparison of the isolates within these groups identified possible transmission events for *E. hormaechei* ST66, ST90, ST108 and ST133 (Fig. 4).

Two separate clusters were detected in the ST66 group, both confined to specific hospitals. The hospital A cluster (purple) was not deemed to reflect transmission as both isolates were taken from the same patient. All isolates from the hospital E cluster (light blue) were predicted to be related, with possible transmission detected in two groups; the first group of isolates (CQS78, CQS73, CQS29, CQS30 and CQS32) differed by less than 4 core SNPs overall and were collected in either March or October 2016. The second group (CQS26 and CQS80) was collected almost a year apart but only differed by 3 core SNPs, indicating probable persistence in the environment. The 13 core SNPs separating these two groups also suggests that direct transmission between these groups of isolates is unlikely, and that transmission from an intermediate source (environmental or unsampled patients/staff) is a more probable explanation.

The ST90 cluster has been described previously [35]. No additional ST90 isolates related to this outbreak were detected in this dataset (CQS46 and MS14389 in reference [35] are derived from the same frozen stock culture in Pathology Queensland). ST108 was found to have three closely related isolates from the same hospital (G; pink), all collected within a 3-month window and probably indicative of transmission. The ST133 group had a cluster of three identical isolates all obtained on the same day (two from the same patient), followed by two more distantly related isolates all from the same hospital. This is likely to indicate short-term transmission between the two patients with identical isolates, with additional long-term transmission or a common reservoir in the environment for at least 10 months.

Four of the five remaining ST groups (Supplementary Data) were found to be unrelated at the core SNP level, suggesting no recent transmission events. This conclusion is also supported by the distinct geographical locations where these isolates were originally obtained. The exception was the ST45 group, which had two isolates from the same hospital. However, due to the core SNP distance (>100 SNPs) and the time between isolation (almost a year apart) they were deemed unlikely to be related to ongoing transmission.

## Discussion

In this study we used WGS to explore the epidemiology of CPE in Queensland. CPE are a major concern for hospitals globally due to their high levels of resistance to antibiotics [37]. Reporting incidences of CPE has become a key priority in combating the spread of these bacteria and understanding their distribution within specific geographical regions. The use of WGS in epidemiological surveillance and nosocomial outbreak investigations has become well established due to its high discriminatory power and reduced costs [38–40]. Short-read sequencing is by far the most commonly used technology due to its high throughput and accurate read quality. However, a major caveat of short-read data is the inability to fully characterize complete plasmid sequences and definitively determine plasmid transmission networks [41]. Here we show how short-read data with selective long-read sequencing was able to characterize the epidemiology of an important plasmid circulating in Queensland.

In our study we found that the majority of CPE in Queensland carry the carbapenemase gene *bla*_IMP-4_, which is consistent with previous reports describing its high prevalence in Australia [11]. This is in contrast to North America, Europe and parts of South East Asia, where *bla*_KPC_ and *bla*_NDM_ have a much higher prevalence [6, 42]. The only cases of uncommon (i.e. non-*bla*_IMP-4_) β-lactamase acquisition in our dataset were associated with recent travel to Greece (for a single *bla*_KPC_ isolate) or India (for all isolates with *bla*_NDM_ variants). These results reflect what has previously been reported by AURA and the CARAlert system [43], where IMP carbapenemases appear to be most common in New South Wales and Queensland, while Victoria has a more even proportion of IMP, NDM and OXA-48-like carbapenemases. This disparity has also been recently characterized in a broader study of CPEs within Victoria, where *bla*_IMP-4_ and *bla*_KPC-2_ were most commonly identified in their cohort [44].

In addition to the high prevalence of *bla*_IMP-4_, presumably carried by a common IncHI2 plasmid, there was also evidence that clonal groups of *E. hormaechei* are simultaneously driving the spread of this carbapenemase gene in Queensland. This was found to be particularly prevalent in intra-hospital transmission events, which could eventually result in community or other healthcare facility spill-over events. While we could not in all cases discern direct transmission events, we found that the number of core SNP differences between isolates with presumed transmission agreed with the SNP threshold defined in a Victorian dataset of CPE, which was ≤23 SNPs for ‘highly likely’ or ‘probable’ local transmission [44]. In this dataset we did not define a specific SNP threshold. This was mainly because of the size of our dataset (<100 isolates), which left few isolates per transmission cluster to draw a conclusive threshold. Defining SNP thresholds can also be problematic as biological variance (e.g. differing environmental pressures), sampling bias (e.g. missing intermediate isolates) and analytical variance (i.e. SNP calling process) can all introduce large inconsistencies in how the thresholds are defined. Future work in the area of transmission clusters is likely to move away from SNP thresholds and towards probabilistic methods of transmission inference [45].

While previous analyses based on traditional molecular techniques were able to observe presumed *in vivo* transfer of *bla*_IMP-4_ [46], the exact mechanism remained unclear. Based on our analysis we suggest that the high prevalence of *bla*_IMP-4_ is likely to be maintained by the widespread dissemination of an IncHI2 plasmid similar to pMS7884A. We also propose that this IncHI2 plasmid has pervaded a broad host range, including all species presented in this study as well as other species, such as *Salmonella enterica* Typhimurium [35, 47]. This adaptability has probably assisted the continued persistence of this plasmid within Australia. Further work is required to determine if this plasmid is able to transfer directly between the species investigated here, or via an intermediate. Another study investigating the presence of carbapenemase genes in silver gulls in south-east Australia revealed a high prevalence of *bla*_IMP-4_ carrying *Enterobacteriaceae* associated with IncHI2 plasmids, presumed to be from gulls feeding on human refuse [48]. This significant environmental reservoir coupled with potentially cyclical re-introduction of this plasmid into human populations could help to explain the endemic levels of *bla*_IMP-4_. Further analysis of human, animal and environmental microbiome and isolate genome datasets from around Australia is needed to fully resolve the missing links in IncHI2/*bla*_IMP-4_ dissemination.

Six isolates from the study were selected for long-read sequencing with Oxford Nanopore MinION to determine the true context of the carbapenemase gene *bla*_IMP-4_. Five isolates were found to carry an IncHI2 plasmid similar to pMS7884A, confirming that this plasmid is probably circulating within Queensland and driving the dissemination of the carbapenemase *bla*_IMP-4_ among different organisms. It is also likely that this plasmid has propagated successfully and widely for a number of years as a consequence of it being able to survive in several different *Enterobacteriaceae *, making it hard to eradicate from environmental reservoirs or detect in patients. Large inversions, insertions or deletions of particular regions appeared to be mainly mediated by insertion sequences. While it was not explored in this study, these genomic rearrangements provide valuable information when discerning plasmid transmission between closely related isolates that may not be detected using short-read sequencing alone. For example, all isolates from the hospital E cluster of ST66 *E. hormaechei* were missing a Tn3-family transposon as well as the tetracycline resistance region. Additionally, all ST830 *E. hormaechei* were missing the streptomycin resistance region, further supporting probable transmission of a single clone between multiple hospitals (Fig. 1 and Supplementary Data). In most cases, the entirety of the ~55 kb MDR region described in pMS7884A was intact, except for CQS51 (where an inversion has caused the region to split) and CQS89 (where an IncL/M plasmid contained some of the MDR region). Several of the isolates were also shown to have gained or lost certain portions of the MDR region, highlighting the plasticity of this region and how easily additional resistance genes could be acquired.

### Conclusion

Using WGS of 107 isolates collected between 2014 and 2017, we show that persistence of the *bla*_IMP-4_ gene in Queensland is associated with the spread of a large IncHI2 plasmid with a broad host range. Long-read sequencing of six example isolates revealed the true context of the *bla*_IMP-4_ gene within IncHI2 plasmids in different species backgrounds and highlighted the ease with which additional mobile regions containing resistance genes are acquired and lost. This genetic plasticity suggests that there is potential for this endemic plasmid to become an even more compelling threat to public health through the acquisition of more potent resistance genes.

## Data Bibliography

1. Roberts, L.W. *et al.* BioProject PRJNA383436 (2017).

2. Partridge, S.R. *et al.* BioProject PRJNA178872 (2012).

## Supplementary Data

Supplementary material 1

Supplementary material 2
