# Supplementary material for: Genomic analysis of carbapenemase-producing Enterobacteriaceae in Queensland reveals widespread transmission of bla IMP-4 on an IncHI2 plasmid
Source: Microb Genom. 2019 Dec 20;6(1):e000321. doi: 10.1099/mgen.0.000321 (PMC7067041; doi:10.1099/mgen.0.000321)
Supplement: Supplementary material 2 [file mgen-6-321-s002.pdf]

Supplementary material

Click here to access/download;Manuscript (Word document);CPE_sup_material_CORRECTIONS.docx

Supplementary Material

Genomic analysis of carbapenemase-producing *Enterobacteriaceae* in Queensland reveals widespread transmission of IncHI2 plasmid

Leah W. Roberts[1,2], Elizabeth Catchpoole[4], Amy V. Jennison[5], Haakon Bergh[4], Anna Hume[4], Claire Heney[4], Narelle George[4], David L. Paterson[6], Mark A. Schembri[1,3], Scott A. Beatson[1,2,3], Patrick N.A. Harris[3,4,6]*

1. School of Chemistry and Molecular Biosciences, The University of Queensland, Brisbane, QLD, Australia
2. Australian Centre for Ecogenomics, The University of Queensland, Brisbane, QLD, Australia
3. Australian Infectious Disease Research Centre, The University of Queensland, Brisbane, Australia
4. Central Microbiology, Pathology Queensland, QLD, Australia
5. Public Health Microbiology Laboratory, Queensland Health Forensic and Scientific Services, Queensland Department of Health, Coopers Plains, QLD, Australia
6. University of Queensland, Faculty of Medicine, UQ Centre for Clinical Research, Royal Brisbane & Women's Hospital, QLD, Australia

CONFIDENTIAL

Table of contents:

Materials and Methods:

- FastANI and MASH comparison of *Enterobacter* isolates
- Phylogenetic analysis of ST830 *E. hormaechei*

Results:

- *bla*$_{\text{NDM}}$ and *bla*$_{\text{KPC}}$ carrying isolates associated with recent travel

Tables:

- **Supplementary Table 1:** CREATE-Q Isolates sequenced to date
- **Supplementary Table 2:** Main β-lactamases found in species other than *Enterobacter*
- **Supplementary Table 3:** Strain list for Supplementary Figure 6

Figures:

- **Supplementary Figure 1:** Complete species tree of carbapenemase-producing Enterobacteriaceae (CPE) from the CREATE-Q study
- **Supplementary Figure 2:** Number of carbapenemase genes per species
- **Supplementary Figure 3:** Number of carbapenemase genes per year
- **Supplementary Figure 4:** Context of *bla*$_{\text{NDM}}$ and *bla*$_{\text{KPC}}$ genes
- **Supplementary Figure 5:** BRIG comparison of CQS20 to *K. pneumoniae* strain KPN528
- **Supplementary Figure 6:** Comparison of *bla*$_{\text{KPC-2}}$ positive isolate CQS9 to ST258 *K. pneumoniae* from Victoria, Australia
- **Supplementary Figure 7:** IncHI2 carrying *Enterobacter* isolates vs. pMS7884A
- **Supplementary Figure 8:** Comparison of non-*Enterobacter* isolates to IncHI2 plasmid pMS7884A
- **Supplementary Figure 9:** Comparison of IncL/M carrying isolates to pEl1573
- **Supplementary Figure 10:** CQS89 carries both IncL/M and IncHI2 plasmid
- **Supplementary Figure 11:** *E. hormaechei* ST830 SNP tree

CONFIDENTIAL

Supplementary Materials and Methods:

FastANI and MASH comparison of *Enterobacter* isolates: As *Enterobacter* isolates in this study were unable to be classified into species using Kraken alone, we used FastANI v1.1 [1] and MASH v2.0 [2] to determine the most likely species by comparison to publicly available *Enterobacter* reference genomes. The reference genomes used were *Enterobacter asburiae* (NZ_CP011863.1), *Enterobacter bugandensis* (NZ_LT992502.1), *Enterobacter clocace* subsp. *cloacae* (NC_014121.1), *Enterobacter hormaechei* subsp. *steigerwaltii* (NZ_CP017179.1), *Enterobacter kobei* (NZ_CP017181.1), *Enterobacter ludwigii* (NZ_CP017279.1), *Enterobacter roggenkampii* (NZ_CP017184.1), *Enterobacter hormaechei* subsp. *xiangfangensis* (NZ_CP017183.1), and *Enterobacter hormaechei* subsp. *hoffmannii* (NZ_CP017186.1).

Unfiltered draft assemblies were compared against the reference genomes using fastANI and MASH (dist) at default settings. The top hit, based on %ANI and greatest number of matching hashes (for fastANI and MASH respectively), was used to identify the species for that isolate.

Phylogenetic analysis of ST830 *E. hormaechei*: SNP distances were determined by mapping trimmed reads from ST830 *E. hormaechei* isolates to the draft assembly for MS7924 using Bowtie (as implemented through Nesoni [3]). Nways produced by Nesoni were interrogated manually to remove low confidence SNPs in repetitive regions. The tree was built using 99 substitutions and RAxML v8.1.15 [4] (1000 bootstraps; GTRGAMMA).

CONFIDENTIAL

Supplementary Results:

bla$_{\mathbf{NDM}}$ **and** ***bla***$_{\mathbf{KPC}}$ **carrying isolates associated with recent travel**

bla$_{NDM}$:

Three isolates were found to carry *bla*$_{NDM}$, including two *E. coli* and one *K. pneumoniae*. The *bla*$_{NDM-6}$ and *bla*$_{NDM-5}$ genes within the *E. coli* isolates (CQS44 and CQS79) were isolated from patients with recent travel to India, and were both carried within composite transposons alongside additional genes with resistance to sulphonamides and tetracyclines (Supplementary Figure 4). Both were found to have a truncated IS*Aba125* insertion sequence upstream of the *bla*$_{NDM}$ gene, which has previously been shown to provide a -35 promoter region resulting in increased transcription of this gene [5]. Comparison of our isolate carrying *bla*$_{NDM-5}$ to all publicly available genomes found a close match to the reference plasmid pLZ135-NDM, isolated in 2012 from an *E. coli* in China (GenBank: MF353156), which harbours the same *bla*$_{NDM}$-carrying transposon flanked by IS*26*. Interestingly, the transposon in this reference appears to be duplicated in tandem (Supplementary Figure 4A). As such, we used read mapping to determine if there was evidence of a similar duplication in our *E. coli* isolates. Depth analysis after read mapping revealed that it is likely that this transposon, carrying the *bla*$_{NDM-5}$ gene, is duplicated in isolate CQS79. There was no evidence for duplication in isolate CQS44.

The *bla*$_{NDM-1}$ gene in *K. pneumoniae* isolate CQS20 appears to be carried within a transposon similar to that found in the reference plasmid pKPN528-1 (GenBank: CP020854), which was isolated from an ST14 *K. pneumoniae* from the USA in 2012 (Supplementary Figure 4B). It is likely that these isolates share a common source in India (based on travel records of both patients) rather than any direct epidemiological link [6]. Nucleotide comparison of the CQS20 draft assembly to pKPN528-1 revealed complete plasmid coverage, indicating that our isolate likely carries a very similar plasmid. As CQS20 was also found to be ST14, we further compared our isolate to the chromosome and two remaining plasmids from the reference *K. pneumoniae* strain KPN528 (GenBank: NZ_CP020853). The chromosome and remaining plasmids from all appeared to have a high level of similarity to our isolate CQS20 (Supplementary Figure 5).

CONFIDENTIAL

bla$_{KPC-2}$:

A single *K. pneumoniae* isolate from our collection (CQS9) was found to carry *bla*$_{KPC-2}$. Nucleotide comparison of the genomic context surrounding this gene found it to be very similar to a plasmid isolated from an ST258 *K. pneumoniae* from Victoria, Australia in 2012 (AUSMDU00008079, GenBank: CP022693) [7]. As our isolate was also found to be ST258, we further compared the chromosome and two other plasmids from AUSMDU00008079 to our isolate. We found that our isolate appeared near identical to the AUSMDU00008079 chromosome (isolated from Victoria in 2012), and retained very similar plasmids (Supplementary Figure 6). This is most likely explained by a common source in Greece (based on recent travel records by our patient) rather than any direct relationship between these isolates, despite the recent report describing circulating *bla*$_{KPC}$+ *K. pneumoniae* in Victoria [7].

The *bla*KPC-2 gene is carried within the mobile transposon Tn*4401a*, which shares 100% nucleotide identity with our isolate. Tn*4401a* is characterised by a 99 bp deletion upstream of the blaKPC-2 gene [8, 9], and is one of the more commonly identified *bla*$_{KPC}$-carrying transposons. Presence of Tn*4401a* has been associated with higher resistance to meropenem, ertapenem and cefepime in *E. coli*, as well as higher *bla*$_{KPC}$ mRNA levels compared to the Tn*4401b* variant [10].

CONFIDENTIAL

Supplementary Tables:

Table 1: CREATE-Q Isolates sequenced to date (Total = 107):

Species	Sequence Types
Enterobacter cloacae complex (n=73)*	
• *Enterobacter asburiae* (n=2)	250
• *Enterobacter cloacae* subsp. *cloacae* (n=1)	167
• *Enterobacter hormaechei* subsp *hoffmannii* (n=1)	145
• *Enterobacter hormaechei* subsp. *steigerwaltii* (n=54)	45, 90, 91, 108, 110, 133, 204, 656, 662, 830, 831
• *Enterobacter hormaechei* subsp. *xiangfangensis* (n=14)	109, 114, 66, 527
• *Enterobacter kobei* (n=1)	191
Enterobacter bugandensis (n=2)	599, NEW
Klebsiella pneumoniae (n=17)**	14, 147, 258, 359, 392, 550, 584, 617, 636, 678, 1122, 1915, 1996
Citrobacter spp. (n=3) ***	185
Klebsiella aerogenes (n=2)	NEW
Klebsiella michiganensis (n=1)	88
Raoultella planticola (n=1)	n/a
Escherichia coli (n=8)	34, 69, 162, 399, 405, 1486, 2851

*Two undetermined/NEW
**Two NEW
***One undetermined

CONFIDENTIAL

Supplementary Table 2: Main β-lactamases found in species other than *Enterobacter*: isolates highlighted in orange appear to have an IncHI2 plasmid similar to pMS7884A, as shown in Supplementary Figure 8.

Isolate	*bla*$_{IMP-4}$	*Bla*$_{SHV}$/*bla*$_{LEN}$	*bla*$_{CTX-M-15}$	*bla*$_{KPC-2}$	*bla*$_{NDM}$	*bla*$_{OXA}$	Other
Raoultella planticola							
CQS82	+		*bla*$_{CTX-M-3}$			OXA-1	*bla*$_{PLA1a}$
Citrobacter amalonaticus							
CQS16	+					OXA-1	
Citrobacter freundii							
CQS88	+	SHV-1	+			OXA-1	*bla*$_{CMY-48}$
CQS51	+	SHV-12	+			OXA-1	*bla*$_{CMY-48}$
Klebsiella aerogenes							
CQS18	+					OXA-1	
CQS5						OXA-48	
Escherichia coli							
CQS12			+		NDM-5		*bla*$_{CMY-42}$
CQS42	+						
CQS44			+		NDM-6	OXA-1	
CQS56	+						
CQS83	+					OXA-1	
CQS70	+						
CQS79			+		NDM-5		*bla*$_{CMY-42}$
MS7925	+	SHV-12					
Klebsiella michiganensis							
CQS33	+	SHV-12				OXA-1	*bla*$_{OXY-1}$
Klebsiella pneumoniae							
CQS90	+	SHV-11	+			OXA-1	
CQS86	+	SHV-187					
CQS84	+	SHV-41					
CQS11		SHV-11	+			OXA-181	
CQS13		SHV-11	+			OXA-181	
CQS20		SHV-11	+		NDM-1	OXA-232, OXA-1	
CQS3		SHV-26				OXA-48	
CQS4		SHV-26				OXA-48	
CQS40		SHV-11	+			OXA-181	
CQS43	+	SHV-26					
CQS57	+	SHV-11					
CQS61	+	SHV-1					
CQS65	+	SHV-187					
CQS71	+	SHV-60					
CQS9		SHV-12		+		OXA-9	
CQS39	+	SHV-41				OXA-1	
CQS62	+	SHV-12, LEN-24				OXA-1	

CONFIDENTIAL

Supplementary Table 3: Strain list for Supplementary Figure 9

Ring number	Strain number	Hospital	Species	Comment
1	CQS5	*B*	*Klebsiella aerogenes*	Missing whole MDR region
2	CQEv1	*A*	*Enterobacter hormaechei* subsp *steigerwaltii*	
3	CQS27	*E*	*Enterobacter hormaechei* subsp *steigerwaltii*	
4	CQS6	*B*	*Enterobacter hormaechei* subsp *steigerwaltii*	Missing whole MDR region
5	CQS60	*Regional*	*Enterobacter hormaechei* subsp *steigerwaltii*	Missing qnrB2 region
6	CQS72	*B*	*Enterobacter hormaechei* subsp *xiangfangensis*	Missing qnrB2 region
7	CQS76	*C*	*Enterobacter hormaechei* subsp *steigerwaltii*	Missing qnrB2 region
8	CQS85	*A*	*Enterobacter asburiae*	
9	CQS89	*Regional*	*Enterobacter hormaechei* subsp *xiangfangensis*	Missing qnrB2 region
10	CQS91	*Regional*	*Enterobacter hormaechei* subsp *steigerwaltii*	
11	CQS42	*A*	*Escherichia coli*	
12	CQS56	*D*	*Escherichia coli*	Missing qnrB2 region
13	CQS70	*B*	*Escherichia coli*	Missing qnrB2 region
14	CQS3	*B*	*Klebsiella pneumoniae*	Missing whole MDR region
15	CQS4	*B*	*Klebsiella pneumoniae*	Missing whole MDR region
16	CQS43	*B*	*Klebsiella pneumoniae*	
17	CQS57	*B*	*Klebsiella pneumoniae*	Missing qnrB2 region
18	CQS61	*E*	*Klebsiella pneumoniae*	
19	CQS65	*Regional*	*Klebsiella pneumoniae*	
20	CQS71	*B*	*Klebsiella pneumoniae*	Missing qnrB2 region
21	CQS84	*Regional*	*Klebsiella pneumoniae*	
22	CQS86	*Regional*	*Klebsiella pneumoniae*	
23	CQS90	*Regional*	*Klebsiella pneumoniae*	Missing qnrB2 region

CONFIDENTIAL

Supplementary Figures:

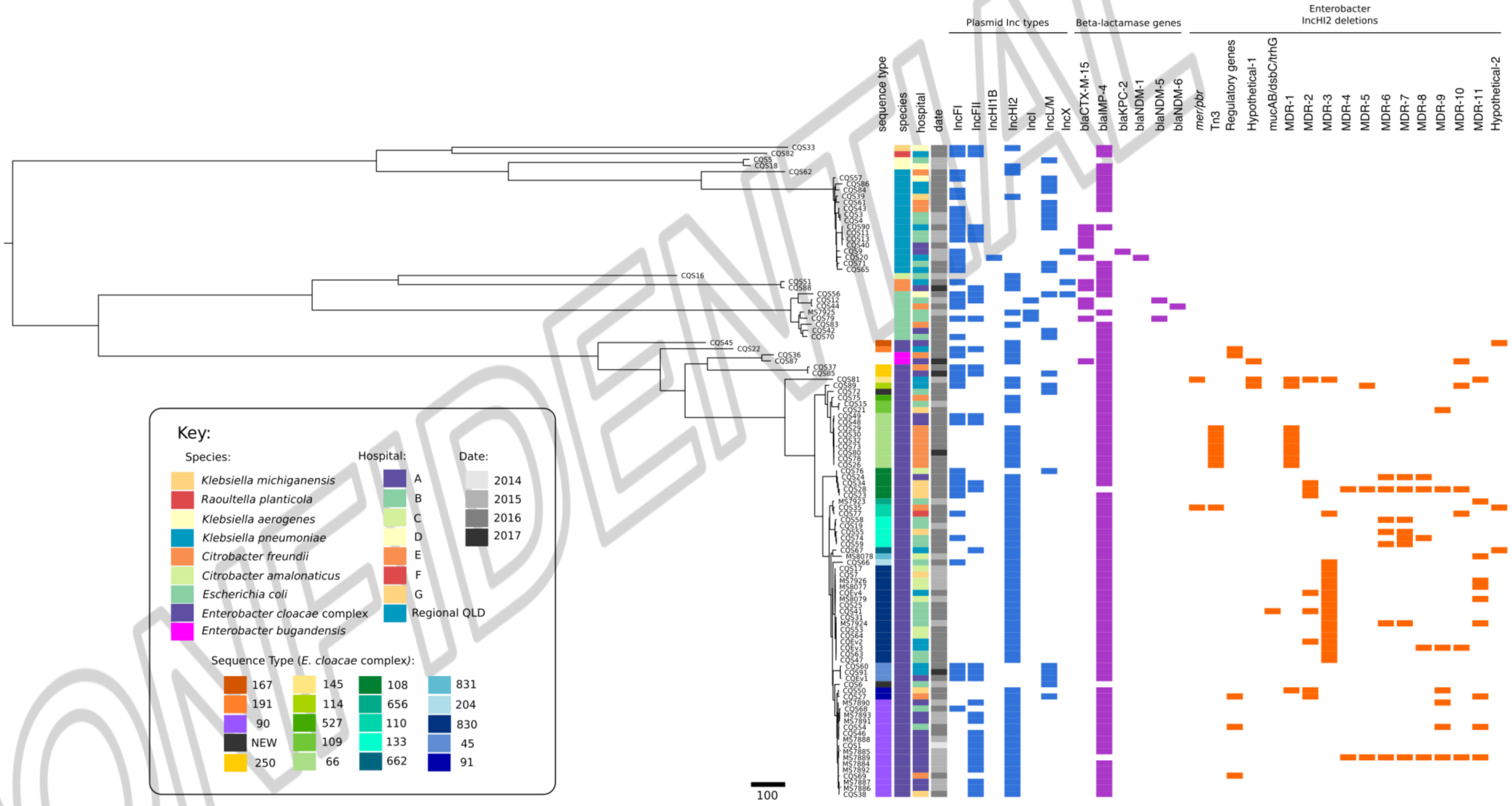

Supplementary Figure 1: Complete species tree of carbapenemase-producing Enterobacteriaceae (CPE) from the CREATE-Q study. Orange blocks represent missing regions identified via comparative analysis of the IncHI2 plasmid in all *Enterobacter* isolates against the representative IncHI2 plasmid pMS7884A (see Figure S5 and Supplementary dataset 2).

CONFIDENTIAL

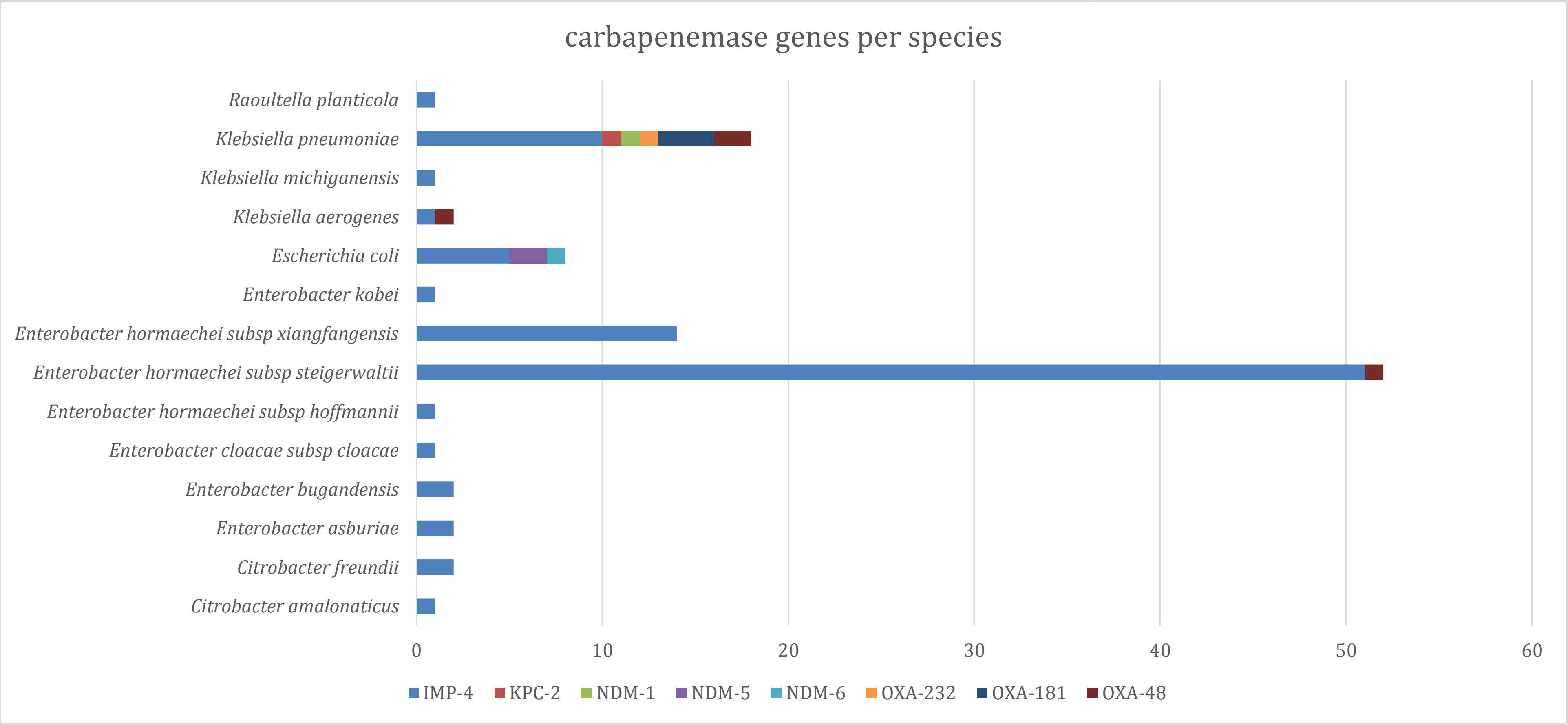

Supplementary Figure 2: Number of carbapenemase genes detected by WGS per species

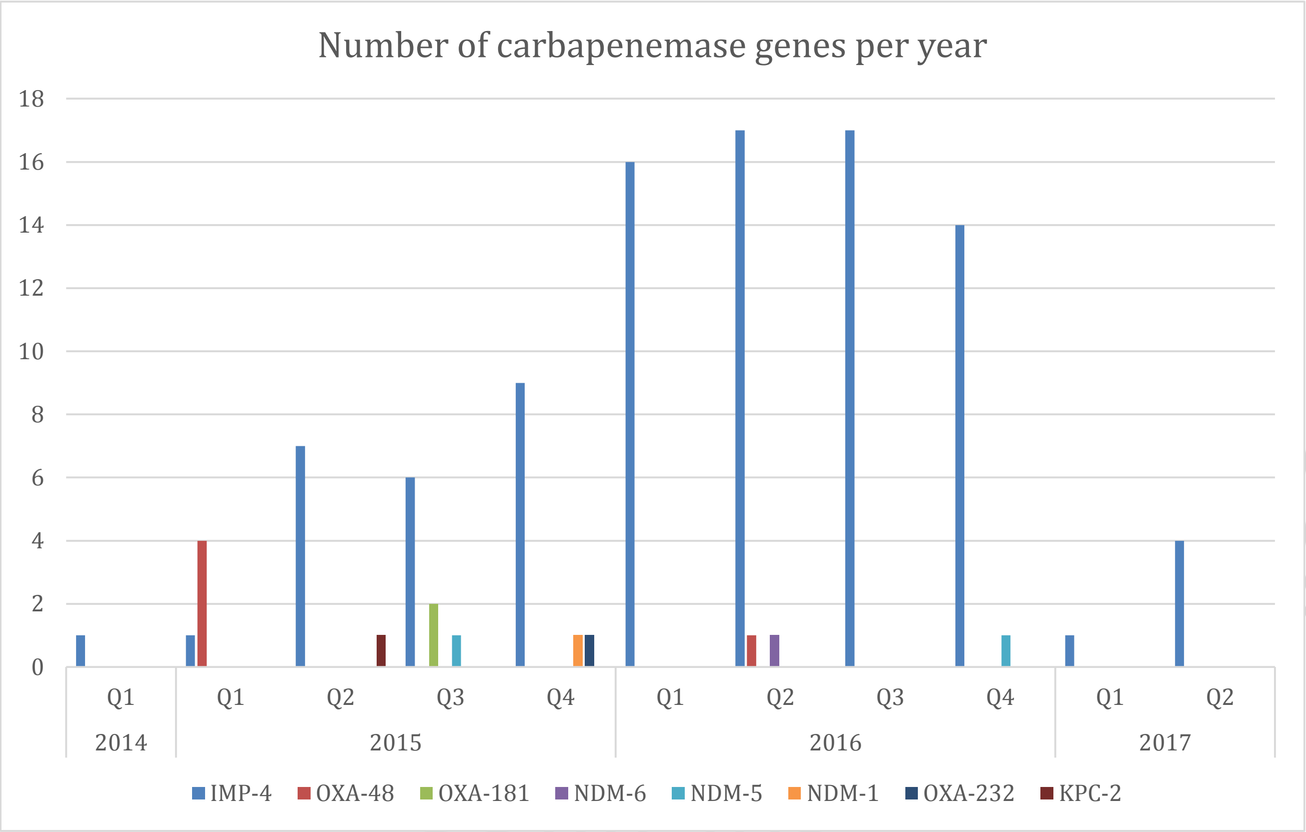

Supplementary Figure 3: Number of carbapenemase genes detected by WGS per year: Q refers to the quarter year (Q1: Jan-Mar, Q2, Apr-Jun, Q3: Jul-Sep, Q4: Oct-Dec).

CONFIDENTIAL

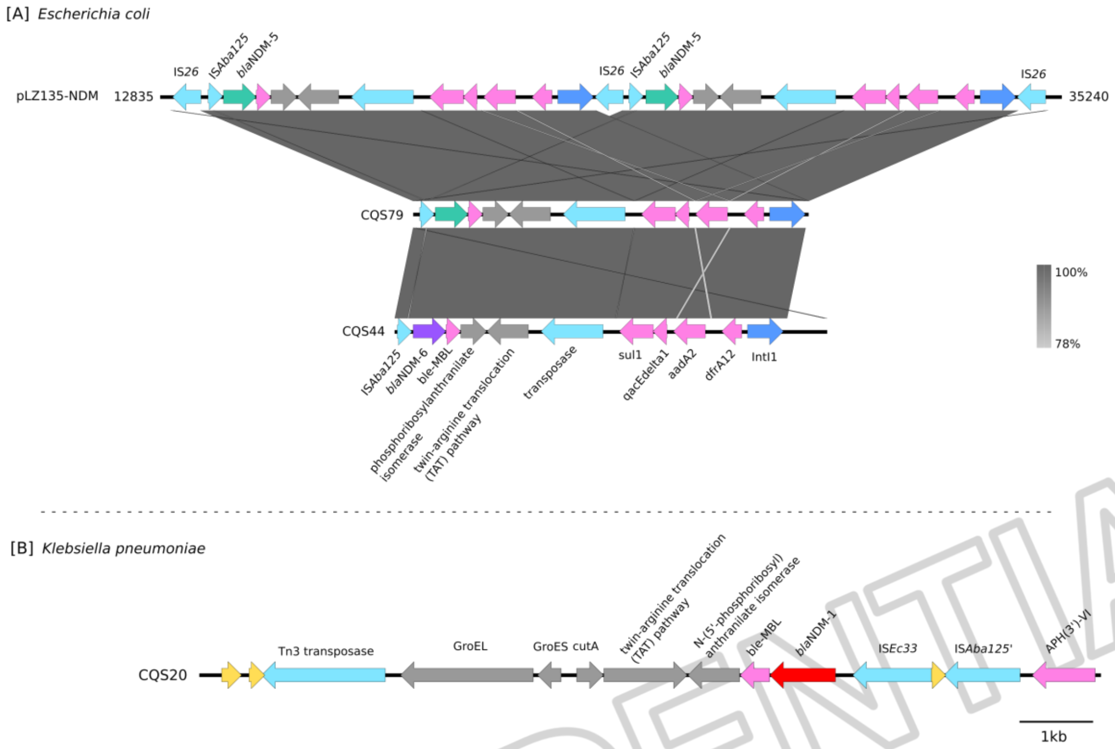

Supplementary Figure 4: Context of *bla*NDM and *bla*KPC genes

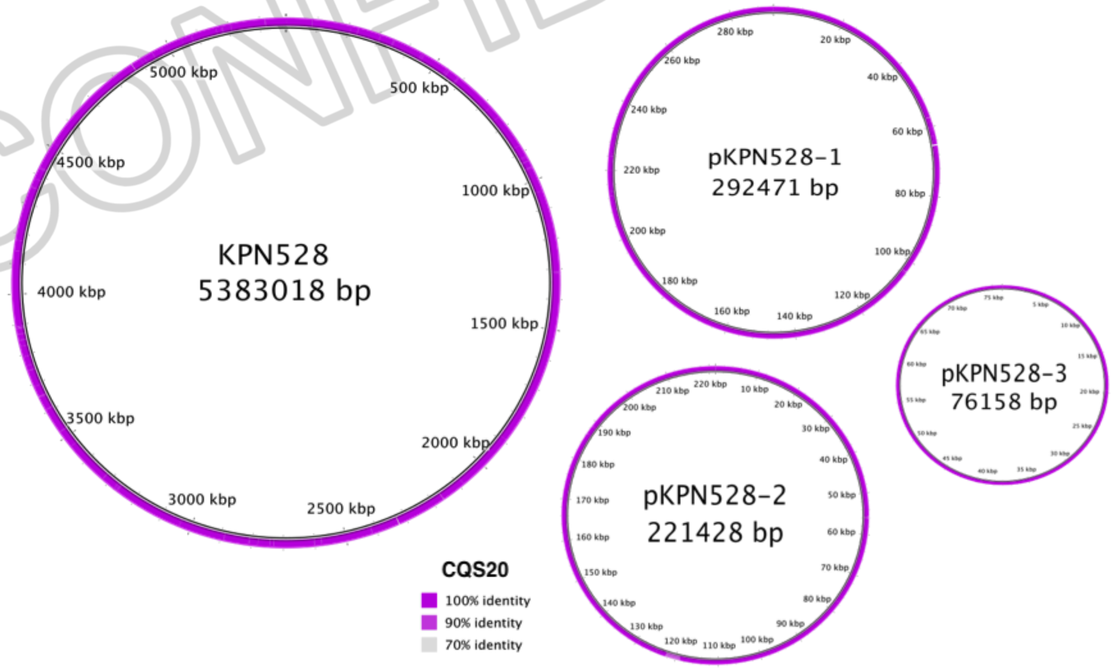

CONFIDENTIAL

Supplementary Figure 5: BRIG comparison of CQS20 to *K. pneumoniae* strain KPN528: Figure was generated using the reference *K. pneumoniae* strain KPN528 (GenBank: CP020853-CP020856) against *K. pneumoniae* isolate CQS20 in BRIG.

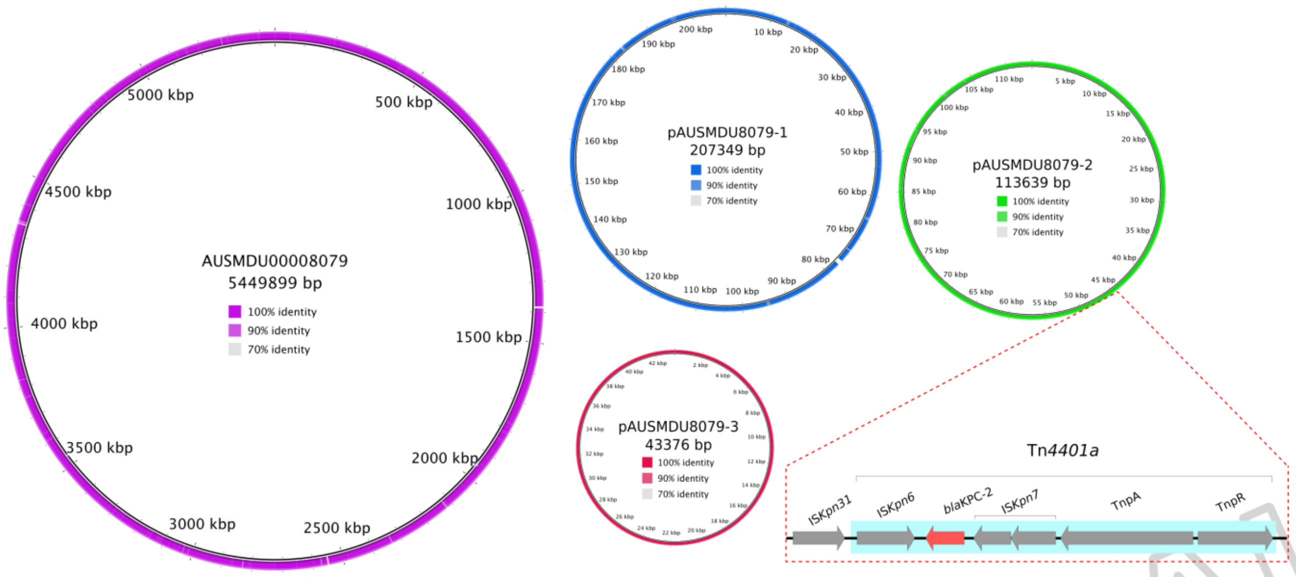

Supplementary Figure 6: Comparison of *bla*$_{KPC-2}$ positive isolate CQS9 to ST258 *K. pneumoniae* from Victoria, Australia [7]

CONFIDENTIAL

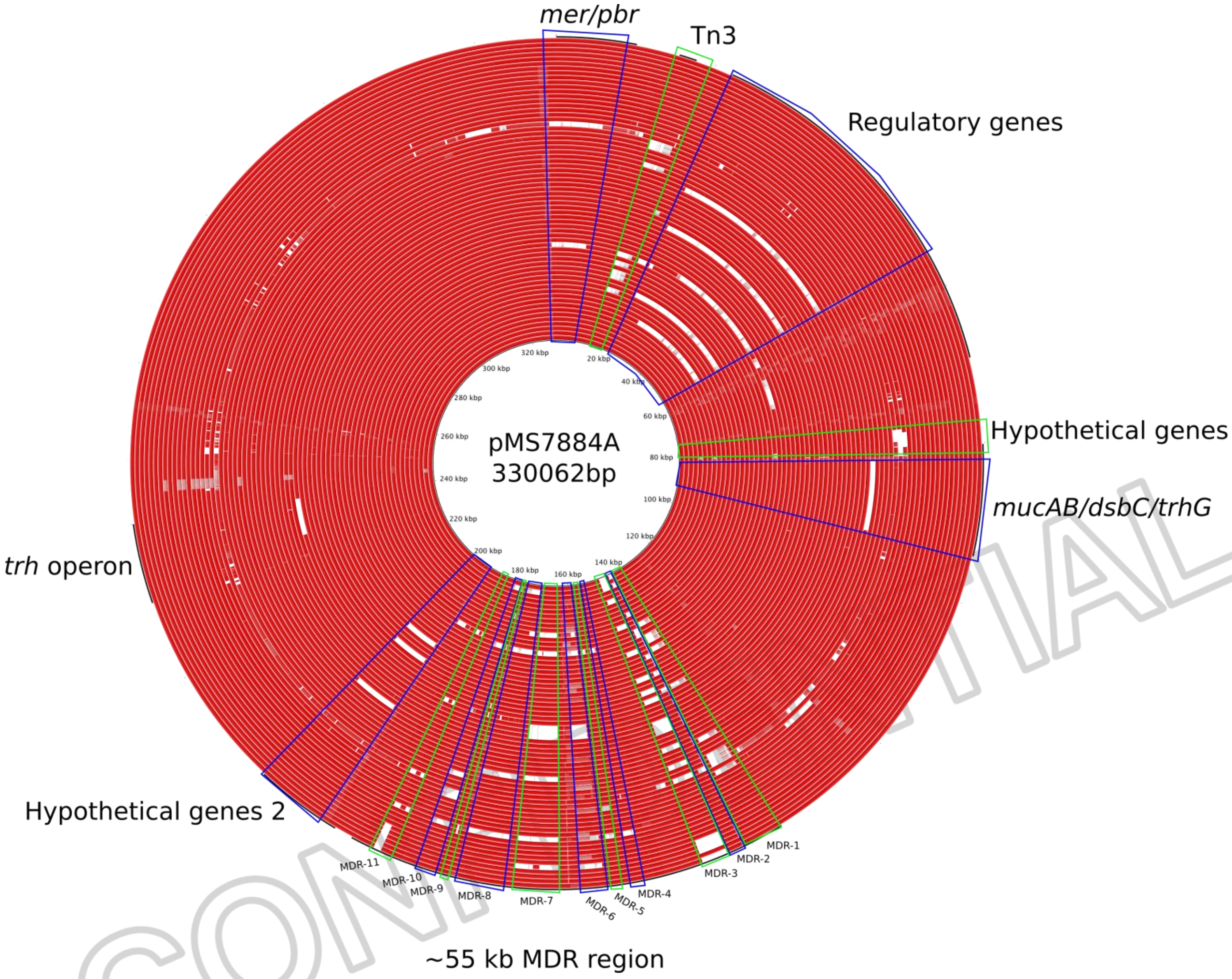

Supplementary Figure 7: IncHI2 carrying *Enterobacter* vs. pMS7884A: Figure was generated using the reference plasmid pMS7884A (GI: 1227395564) against all IncHI2+ *Enterobacter* draft assemblies from our study (n=65/75). Outer annotated regions correspond to regions missing in particular isolates. Regions blocked in blue/green outline represent missing regions mapped to Figure S1. See supplementary dataset 2 for complete list of isolates and further description of missing regions.

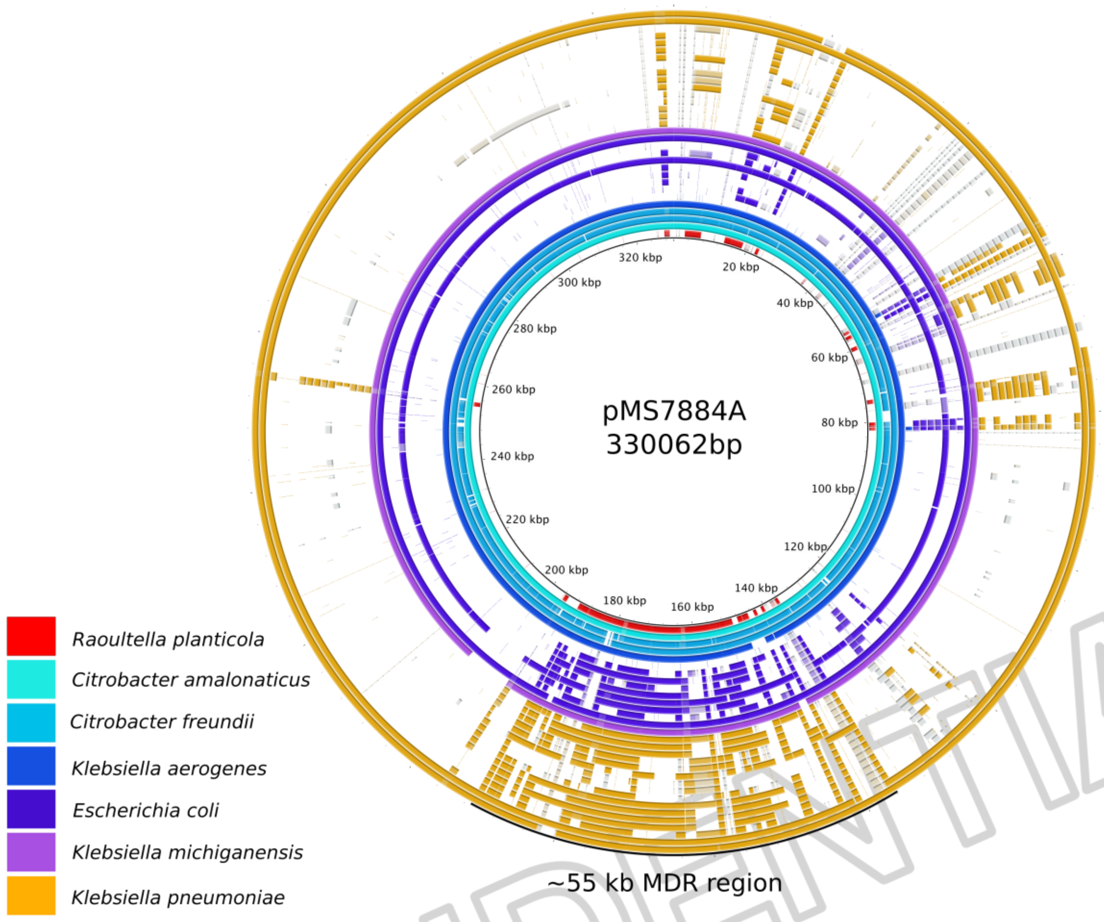

Supplementary Figure 8: comparison of non-*Enterobacter* isolates to IncHI2 plasmid pMS7884A (see Supplementary Table 2 for specific isolate details). Figure was generated using the reference plasmid pMS7884A (GI: 1227395564) against all non-*Enterobacter* draft assemblies from our study.

CONFIDENTIAL

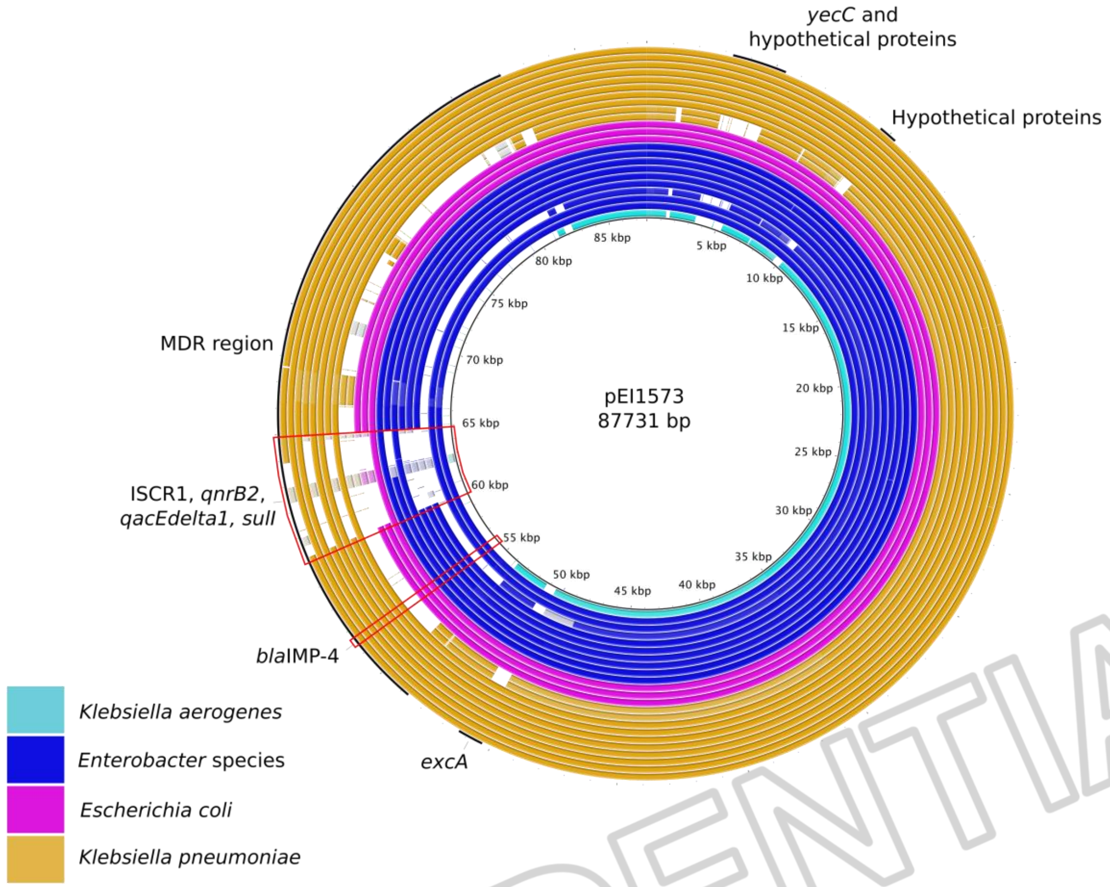

Supplementary Figure 9: Comparison of IncL/M carrying isolates to pEl1573: Figure was generated using the reference plasmid pEl1573 (GI: 414340551) against all IncL/M+ isolate draft assemblies from our study. Supplementary Table 3 contains a list of isolates from inner to outermost ring.

CONFIDENTIAL

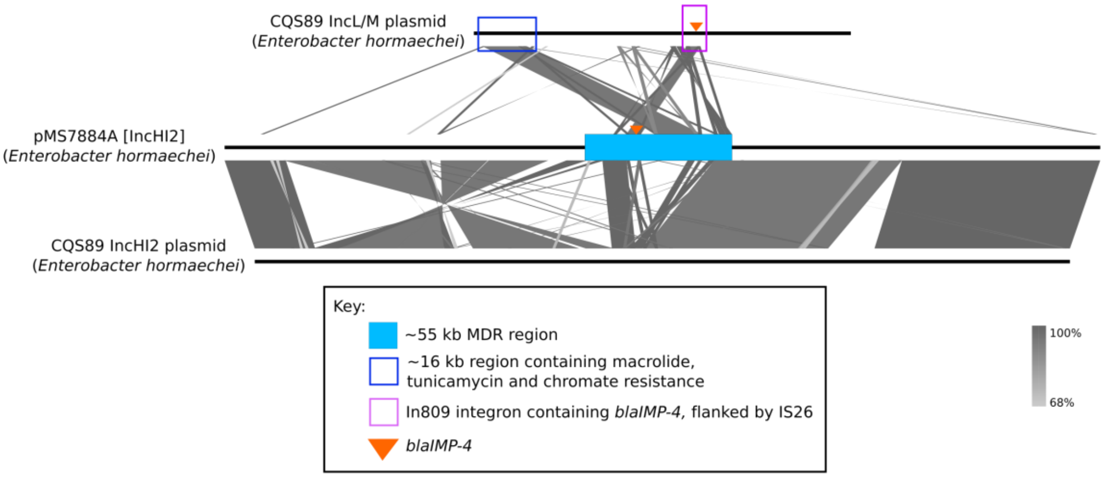

Supplementary Figure 10: CQS89 carries both IncL/M and IncHI2 plasmid

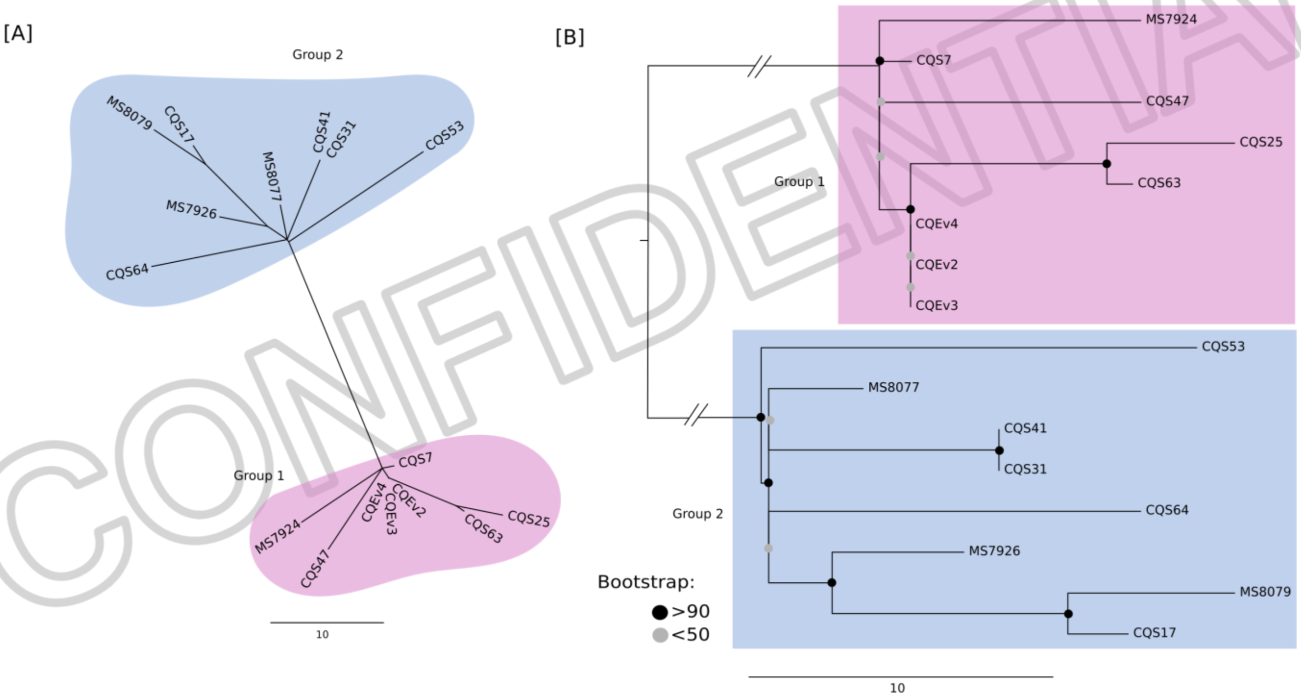

CONFIDENTIAL

Supplementary Figure 11: *E. hormaechei* ST830 SNP tree: Trimmed reads were mapped to the draft assembly reference MS7924 (*E. hormaechei*) using Bowtie (as implemented in Nesoni). Core SNPs were curated manually to remove low-confidence SNPs and SNPs in repetitive regions. Remaining SNPs were used to create the final tree in RAxML (1000 bootstrap, GTRGAMMA mutation rate, seed value 456). [A] unrooted, [B] mid-point rooted.

References:

1. Jain, C., et al., *High throughput ANI analysis of 90K prokaryotic genomes reveals clear species boundaries.* Nat Commun, 2018. **9**(1): p. 5114.
2. Ondov, B.D., et al., *Mash: fast genome and metagenome distance estimation using MinHash.* Genome Biol, 2016. **17**(1): p. 132.
3. Victorian-Bioinformatics-Consortium. *Nesoni*. 2014 [cited 2016; v 0.130:[Available from: https://github.com/Victorian-Bioinformatics-Consortium/nesoni.
4. Stamatakis, A., *RAxML version 8: a tool for phylogenetic analysis and post-analysis of large phylogenies.* Bioinformatics, 2014. **30**(9): p. 1312-3.
5. Partridge, S.R. and J.R. Iredell, *Genetic contexts of blaNDM-1.* Antimicrob Agents Chemother, 2012. **56**(11): p. 6065-7; author reply 6071.
6. Long, S.W., et al., *Population Genomic Analysis of 1,777 Extended-Spectrum Beta-Lactamase-Producing Klebsiella pneumoniae Isolates, Houston, Texas: Unexpected Abundance of Clonal Group 307.* MBio, 2017. **8**(3).
7. Kwong, J.C., et al., *Translating genomics into practice for real-time surveillance and response to carbapenemase-producing Enterobacteriaceae: evidence from a complex multi-institutional KPC outbreak.* PeerJ, 2018. **6**: p. e4210.
8. Gootz, T.D., et al., *Genetic organization of transposase regions surrounding blaKPC carbapenemase genes on plasmids from Klebsiella strains isolated in a New York City hospital.* Antimicrob Agents Chemother, 2009. **53**(5): p. 1998-2004.
9. Perilli, M., et al., *Emergence of blaKPC-3-Tn4401a in Klebsiella pneumoniae ST512 in the municipal wastewater treatment plant and in the university hospital of a town in central Italy.* J Glob Antimicrob Resist, 2013. **1**(4): p. 217-220.
10. Cheruvanky, A., et al., *Enhanced Klebsiella pneumoniae Carbapenemase Expression from a Novel Tn4401 Deletion.* Antimicrob Agents Chemother, 2017. **61**(6).

CONFIDENTIAL
